# Agarose-Based Biomaterials: Opportunities and Challenges in Cartilage Tissue Engineering

**DOI:** 10.3390/polym12051150

**Published:** 2020-05-18

**Authors:** Mohammad Amin Salati, Javad Khazai, Amir Mohammad Tahmuri, Ali Samadi, Ali Taghizadeh, Mohsen Taghizadeh, Payam Zarrintaj, Josh D. Ramsey, Sajjad Habibzadeh, Farzad Seidi, Mohammad Reza Saeb, Masoud Mozafari

**Affiliations:** 1 Polymer Engineering Department, Faculty of Engineering, Urmia University, Urmia 5756151818, Iran; salati.mohammadamin1999@gmail.com (M.A.S.); jkhazaei78@gmail.com (J.K.); pmbltzr@gmail.com (A.M.T.); ali.samadi2@gmail.com (A.S.); 2 Center of Excellence in Electrochemistry, School of Chemistry, College of Science, University of Tehran, Tehran 11155-4563, Iran; clarion.payam@gmail.com (A.T.); m.taghizadeh@gmail.com (M.T.); 3 School of Chemical Engineering, Oklahoma State University, 420 Engineering North, Stillwater, OK 74078, USA; josh.ramsey@okstate.edu; 4 Department of Chemical Engineering, Amirkabir University of Technology (Tehran Polytechnic), Tehran 1591639675, Iran; sajadhabibzadeh@gmail.com; 5 Provincial Key Lab of Pulp and Paper Science and Technology and Joint International Research Lab of Lignocellulosic Functional Materials, Nanjing Forestry University, Nanjing 210037, China; f_seidi@njfu.edu.cn; 6 Department of Resin and Additives, Institute for Color Science and Technology, Tehran P.O. Box 16765-654, Iran; 7 Department of Tissue Engineering and Regenerative Medicine, Faculty of Advanced Technologies in Medicine, Iran University of Medical Sciences, Tehran 144961-4535, Iran

**Keywords:** agarose, biomaterials, hydrogels, cartilage, tissue engineering, regenerative medicine

## Abstract

The lack of adequate blood/lymphatic vessels as well as low-potential articular cartilage regeneration underlines the necessity to search for alternative biomaterials. Owing to their unique features, such as reversible thermogelling behavior and tissue-like mechanical behavior, agarose-based biomaterials have played a key role in cartilage tissue repair. Accordingly, the need for fabricating novel highly efficient injectable agarose-based biomaterials as hydrogels for restoration of injured cartilage tissue has been recognized. In this review, the resources and conspicuous properties of the agarose-based biomaterials were reviewed. First, different types of signals together with their functionalities in the maintenance of cartilage homeostasis were explained. Then, various cellular signaling pathways and their significant role in cartilage tissue engineering were overviewed. Next, the molecular structure and its gelling behavior have been discussed. Eventually, the latest advancements, the lingering challenges, and future ahead of agarose derivatives from the cartilage regeneration perspective have been discussed.

## 1. Introduction

Millions of people in the world are suffering from articular cartilage diseases (ACD) and its side effects, such as anterior cruciate ligament (ACL) and osteochondral defects (OCD). It is expected that OCD will be the principal reason for incapacity in the next decades, with 35% of the population affected. The occurrence of OCD depends on age, gender, and environmental conditions. Although treating the defects using surgeries is promising, cartilage regeneration remains as a post-surgery complication because of its structural complexity and low metabolic performance [1,2]. Furthermore, the long-term healing period of internal articular cartilage demands new strategies for repairing cartilage without surgery [3].

Tissue engineering is an interdisciplinary branch of science for designing and implementing novel treatment strategies based on natural/synthetic materials along with cell and therapeutic agents to treat the injured tissues [4,5,6]. Tissue engineering has opened a promising path to regenerate the cartilage using modified natural/synthetic biomacromolecules. The first step in material selection for tissue engineering is the tissue recapitulation by the selected scaffold [7,8,9]. Therefore, the mechanical behavior of the intended material should match with its nearby cartilage in order to keep its function [10,11]. Biopolymers such as chitosan [12,13], alginate [14,15], gelatin [16,17], and agarose [18] have been widely used for tissue engineering [19,20] because of their proper biocompatibility, controllable degradability, and tissue-mimicking performance. 

**Figure 1:**
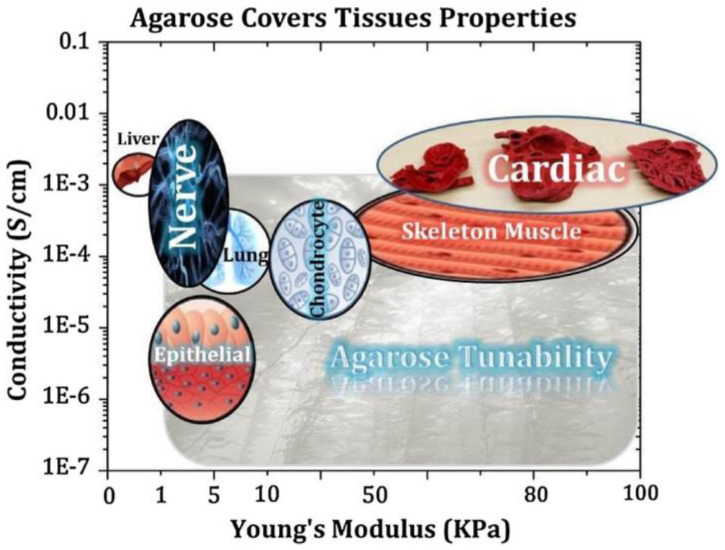
Adjustable features of agarose can result in flexible characteristics similar to cells and tissues. Here, the conductivity and Young’s modulus of agarose-based biomaterials are patterned. Reprinted with permission from [18].

Agarose has been used vastly in biomedical applications because of its controlled self-gelling properties, water-solubility, adjustable mechanical properties, and non-immunogenic properties. Agarose, based on its stiffness and functional groups, can support cellular adhesion, proliferation, and activity. Agarose has adjustable water adsorption capacity, which provides the cells with a suitable microenvironment for cellular activity [21]. Agarose-based hydrogels have considerably been utilized in the investigation of mechanical load reaction for chondrocytes and mesenchymal stem cells (MSCs) [22]. Agarose has acquired a wide range of applications such as drug delivery [23,24], cancer therapy [25,26], tissue engineering and regenerative medicine [27,28,29,30], and disease diagnosis, controlling, and treatment [31,32,33]. It has been utilized in various tissue engineering applications, including the neural system [34], bone formation [35], cardiac regeneration, wound healing, and attachment to tissues such as skin [36], brain [37], and cornea. The properties of agarose are tunable by concentration, functionalization, and blending to mimic the desired tissue performance. Figure 1 exhibits the agarose gel performance for various tissues and cells.

## 2. Cartilage Types, Properties, and Formation

Cartilage is a smooth elastic tissue, which covers the end of bones at the joints and protects them against mechanical stresses. There are three types of cartilage: articular (hyaline), fibrous, and elastic [38,39]. Heterotypic collagen II/IX/XI fibrils and proteoglycan–glycosaminoglycan networks of aggrecan and hyaluronan are the dominant structures of the cartilage extracellular matrix (ECM). Hyaline is an elastic, flexible, and wear-resistant tissue that is accessible inside the joint to carry and disrupt the weight. The cartilages of the ear, larynx, and epiglottis are more elastic than the hyaline. Fibrocartilage as inexorable cartilage is available in the knee and between the vertebrae [40].

**Figure 2:**
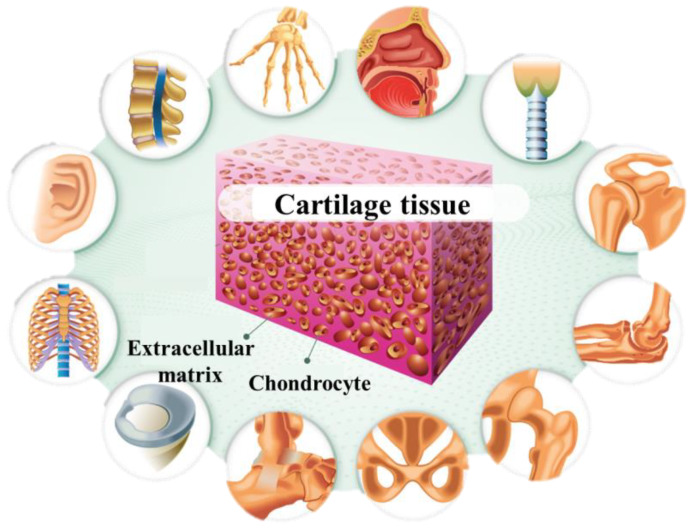
Schematic of the cartilage tissue structure. (a) Collagen (type II) and chondrocytes are two major components of cartilage tissue, the application of cartilage tissue in different parts of the human body (reproduced with permission from [42]).

Low-speed regeneration of cartilage tissue is chiefly because of a lack of blood vessels and nerves. This phenomenon may lead to rheumatoid arthritis, inflammatory disease, and deterioration of the joints, causing pain for a long time. Collagen (type II), hyaluronan, and dormant sulfate are the major components of the cartilage tissue ECM. Cartilage damage is accompanied by ECM degradation and mobilization of joint chondrocytes from other areas with decreased infiltration and vascularization of inflammatory cells. Figure 2 shows the cartilage structure [41].

Different biomaterials can be utilized in repairing the cartilage; however, we should point out that the signal paths and biological signs involved in the cartilage are activated so that we can begin to interact and reconstruct the muscles. Chondrogenesis is a process by which various cellular signals are sent in the regulation of cartilage formation [43]. Multiple factors have been announced for cartilage repair, such as transforming growth factor β (TGFβ), parathyroid hormone-related protein (PTHrP), Wnts, Indian hedgehog, thyroid hormone, bone morphogenetic protein (BMP) superfamily, platelet-derived factors (PDGFs), insulin-like growth factors (IGFs), fibroblast growth factors (FGFs), and different vitamins (Table 1) [44]. TGFβs play a vital role in regulating the proliferation/differentiation of chondrocytes. TGFβ stimulates the RY-box expression causing the formation of collagen type II and aggrecan in the early condensation phase of MSCs [45]. Wnt and β-catenin-dependent signaling pathways possess vital functions in developing chondrocytes (Table 2). The β-catenin-dependent stimulates endochondral ossification and axial growth [44].

## 3. Agarose Properties

**Figure 3:**
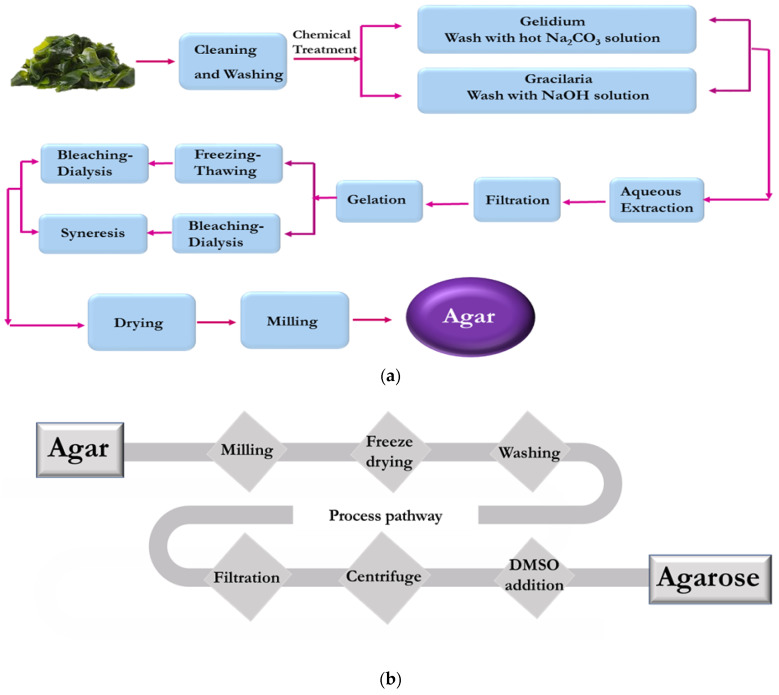
Schematic illustration of extraction route for the production of (**a**) agar from algae through a chemical treatment and physical filtration for (**b**) agarose from agar source using DMSO solution.

Extraction of agar from the algae source [46], Ahnfeltia plicata [47], Gelidium amansii [48], and Eucheuma [49], using chemical treatment (NaOH [50], ionic liquids [51], Na_2_CO_3_ [50]), is the main method conducted, as illustrated by the steps detailed in Figure 3. Agar was utilized as a source of agarose extraction, in the same way as has been explained above [46,48] (see Figure 1).

The molecular weight of agarose is almost 120,000 g/mol (12 kDa). It is in the form of a white powder that dissolves in hot water to form a gel when cooled to below an upper critical solution temperature (UCST) [18]. Furthermore, agarose dissolves in several organic solvents such as dimethyl sulfoxide (DMSO), dimethylformamide (DMF), formamide (FA), N-methylformamide (MFA), and 1-Butyl-3-methylimidazolium chloride (BmimCl) [52,53]. Agarose is a part of the agar that is obtained by separating the agar–peptide. Various factors such as molecular weight, concentration, and lateral groups significantly affect the melting and gelling temperature [19,52,54]. Overall, 1.5% of each agarose type, such as types II, VII, and IX-A, have different gelling and melting temperatures, at 26, 26–30, and 17 °C, respectively [55].

**Figure 4:**
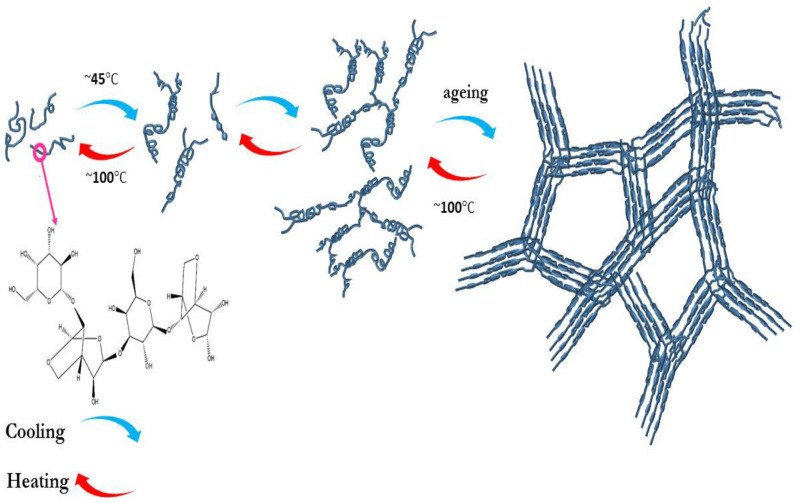
The molecular structure of agarose and schematic of its gelling process (reprinted from [21]).

The agarose gelation process is divided into three stages: induction, gelation, and quasi-equilibrium [56,57]. The mechanism of gelation is usually characterized using rheological experiments [56], and it includes nucleation and growth of nuclei. Several agarose nuclei are formed in the first step followed by the growing of nuclei, which gradually form agarose-rich networks (Figure 4). In agarose, the formation of gel is related to the agarose molecule twisting linked to the transplantation of hydrogen and electrostatic penetration [58]. In the end, it is essential to note that the concentration of the agarose gel determines its permeability [59].

A big challenge in cartilage tissue engineering is to achieve mechanical properties similar to cartilage itself [60]. Agarose hydrogels can be an appropriate selection for cartilage tissue engineering because of the adjustable mechanical properties, swelling ratio, and therapeutic factors. The mechanical properties of agarose can be tuned by altering the concentration. For instance, the elastic modulus for different concentrations of agarose with compression membranes has been calculated from 0.5%, 1.0%, 2.5%, and 5.0% of the agarose gel as 5.3, 38, 254, 929 KPa, respectively [61].

Comparing Young’s modulus of different body organs with agarose is of particular interest in tissue engineering (Figure 1), but on the other hand, Young’s modulus of agarose is less than 0.5 MPa [62,63], indicating that agarose has high-pressure tolerance compared to natural tissues. Young’s moduli for bone, cartilage, nerve, cardiac, skeletal muscle, endothelium, liver, and lung are in the range of 1–20 GPa [64], 10–20 KPa [65], 0.1–2 KPa [66], 30–400 KPa [67], 20–100 KPa [68], 1–7 KPa [69], 0.3–0.8 KPa [70], and 1–5 KPa, respectively [70].

The mechanical behavior of agarose is comparable to articular cartilage upon static or dynamic loading [71,72]. Since cartilage and agarose are both hydrated materials, they exhibit strain-dependent hydraulic permeability, which means that resistance to water transportation through the hydrogel increases with the increase of applied deformation (and the collapse of the pores) [73].

## 4. Agarose in Cartilage Regeneration

Cartilage exhibits arduous regeneration because of its avascular nature. Therefore, cartilogenesis is known as a challenging field in regenerative medicine and tissue engineering. Cartilage as a microenvironment possesses a hypoxic condition that facilitates the differentiation of MSCs to chondrogenesis rather than osteogenesis [74]. The degree of success in cartilage tissue engineering is pertinent to the cells, growth factors, and biomaterials. Moreover, the age and size of the injured section impacts the regeneration process. Biomaterials in cartilage repair, along with proper biocompatibility and biodegradability, should offer a suitable milieu for proliferation, differentiation, and migration of chondrocytes. Guaccio et al. revealed that cartilage growth is usually diminished by the problem of inadequate oxygen/nutrient source to cultured cells in 3D platforms. It was understood that oxygen utilization level is two times greater in agarose compared to collagen, indicating that the nature of the material greatly impacts cell metabolic performance [75]. Applying an agarose hydrogel-based scaffold for cartilage regeneration stabilized the chondrocyte phenotype and enhanced the proteoglycan and glycosaminoglycans precipitation [76]. Cigan et al. encapsulated the human chondrocyte in an agarose scaffold for cartilage regeneration. Engineered cartilaginous structure substantially appeared like native human cartilage, and possessed compressive Young’s and dynamic moduli of about 250 and 950 kPa, respectively. Moreover, it was composed of 5.7% (w/w) of glycosaminoglycans and 1.5% (w/w) collagen [76]. Implantation of autologous chondrocyte is a recognized technique for symptomatic articular flaws. Selmi et al. used agarose-based hydrogel to enhance cell phenotypic constancy and improve surgical management. Overall, seventeen patients were used as clinical trials and were surveyed for two years. Clinically, all the patients were treated substantially. Patients with defects more than 3 cm^2^ were improved drastically compared to those with smaller ones. Moreover, in eight cases, dominant hyaline cartilage-like repair tissue was detected [77]. Kock et al. studied the agarose concentration on chondrocyte behavior [19]. Matrix formation around the chondrocyte was affected by agarose concentration. A low concentration of agarose facilitated the proteoglycans diffusion. Agarose with 1% concentration resulted in collagen II deposition in a radial manner, while 2% and 3% agarose concentrations resulted in a dense layer of collagen formation around the cell. The 1% agarose contained more proteoglycans compared to those having 2% and 3% agarose. It was deduced that the agarose concentration reduction caused more nutrient diffusion and enhancement of matrix deposition like collagen fibril. At lower concentrations of agarose, a uniform matrix was developed that increased the modulus at equilibrium state. Therefore, low concentration agarose increased the deposition of the extracellular matrix, which improved the mechanical properties [60]. In Table 3, the mechanical properties, the concentrations and the methods of applications of agarose-based biomaterials are presented.

Agarose blending enhances its cellular activity and regeneration. Singah et al. blended the agarose with silk to regenerate the cartilage [53]. The results indicated that the silk/agarose scaffold increased sulfated glycosaminoglycans (sGAG) and collagen deposition as a signature of the preservation of chondrogenic phenotype. Moreover, it was revealed that up-regulation of cartilage-specific aggrecan, sox-9 (∼1.5-fold) and collagen type II (∼2-fold) marker genes (*p* ≤ 0.01) in blended hydrogels collagen and fibronectin colocalization was a suitable method to improve the agarose microenvironment for chondrocytes culture. Furthermore, the connection between collagen or fibronectin with chondrocytes and the connection between chondrocytes with living cartilage resulted in an increasing matrix and cohesion of matrix–matrix that improved cartilage regeneration [70]. Platelet-rich plasma (PRP) is the source of known growth factors. Thus, a scaffold was made using a platelet-rich plasma–agarose gel, and the results showed that PRP–agarose gel was extensively uniform in the structure with high-level concentration, as a signal of an increased amount of collagen type -I mRNA expression in the structure [79]. Dekosky et al. synthesized an interpenetrating network of hydrogels based on agarose/Poly(ethylene Glycol) to encapsulate cells for cartilage regeneration [81]. Espinosa et al. synthesized a magnetic fibrin–agarose hydrogel for cartilage regeneration. It was noticed that magnetic particles increased the mechanical features of fibrin–agarose hydrogels. Moreover, chondrocytes seeded in magnetic substrate expressed type II collagen. Magnetic hydrogels demonstrated a high ability for hyaline cartilage-like tissue engineering [82].

**Figure 5:**
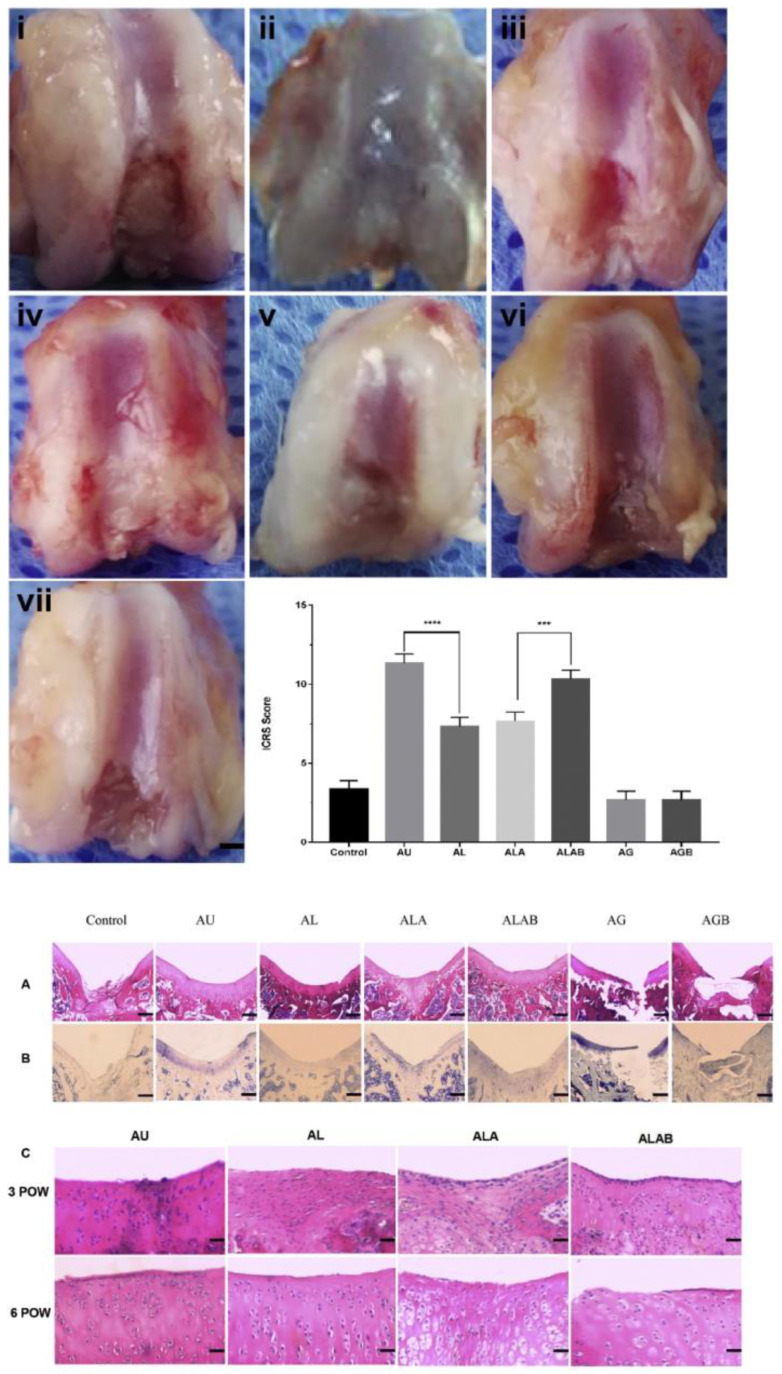
cartilage injury after transplantation of different groups: (**i**) nontransplant control, (**ii**) autologous articular cartilage (AU), (**iii**) allogeneic (same species, different rat) articular cartilage (AL), (**iv**) allogeneic articular cartilage replacing agarose gel (ALA), (**v**) allogeneic articular cartilage replacing agarose gel with bFGF (ALAB), (**vi**) agarose gel (AG), and (**vii**) agarose gel with bFGF (AGB). The cartilage restoration evaluation was drastically greater in the AU group than that in the AL group, as was the ALAB group compared with the ALA group. Assessment of histological data after transplantation. (**A**,**B**) Nontransplant control: amorphous reparative tissue filling the subchondral region. AU: intensive staining covering the defect. AL: the intensity of staining in the regenerated region was less than that of AU and ALAB. ALA: partly positive cartilage organization in the area. ALAB: amounts of cartilage-like tissue restored in the full-thickness defect. AG and AGB showed the agarose gel occupied the space and hindered the reconstructive process. (**C**) Histological findings in knee cartilage in the transplantation site at postoperative week (POW) 3 and POW 6 (hematoxylin and eosin). At POWs 3 and 6, the ALAB and AU groups showed no obvious evidence of rejection. In the other two groups, chondrocytes with small, condensed nuclei were visible at each time point. Scale bar is 50 μm for all images (Reprinted with permission form [83]).

Osteochondral flaws or cartilaginous damage can be treated using allogeneic cartilage transplantation. Immune rejection is a challenging issue with grafting, though. Yang et al. used basic fibroblast growth factor (BFGF) and agarose to regenerate the cartilage with minimum immunological stimulation. Treated with BFGF, agarose and allogeneic cartilage resembled the autologous one. The monocytes level in allografts was at its maximum level in the spleen and blood; the CD4+ T cells number in the allogeneic group was greater than others. Allogeneic cartilage transplantation stimulates acute immune rejection, which deals with the authenticity of the implant. The combination of BFGF and agarose simplifies the aim of immune privilege and enhances the allograft tissues’ performance (Figure 5) [83].

## 5. Future Perspective and Concluding Remarks

Agarose is a wonderful biopolymer extracted from agar possessing extraordinary properties such as excellent porous structure with interconnected pores to ease the nutrient, oxygen permeation, and waste exchanges, the desired biodegradability and biocompatibility, a high ability to mimic human tissue, perfect cell/matrix interaction, and great hydrophilicity and elasticity. Such promising characteristics allow the migration/proliferation of cells, and a wide range of Young’s modulus associated with ionic conductivity makes agarose a supreme applicant for the regeneration of many human organs. The biggest advantage of agarose hydrogels is the encapsulation of chondrocytes and cartilage cells at a cellular level which enables 3D cultures missing the loss of phenotype or morphology. Cartilage tissue due to the absence of ample blood vessels and a low amount of stem cells requires a suitable environment like hydrogels for filling the defected part when it gets damaged and makes a human-tissue-like environment to help the regeneration process. Agarose and its composites presented a conspicuous role in the regeneration of cartilage tissue. Distinctive characteristics of agarose-based hydrogels such as excellent mechanical properties which can mimic the real hard/soft tissue with high agreement, non-immunological, great biocompatibility, nontoxicity, and high cell/matrix interaction have emphasized their application in cartilage repair research. The concerns and studies to fabricate the best compatible agarose-based hydrogels for engineered tissue cartilage applications and the development of complexes and interlinked types of agarose hydrogels have acquired more currency amongst scientists. However, these hydrogels still need more advanced assessments in clinics and real-world applications. Consequently, an additional organized attempt is crucial to filling the current gap in cartilage repair that involves details of the main elements of agarose-based hydrogels. Moreover, thanks to novel techniques such as bioprinting, the novel and precise 3D-printed scaffold can be fabricated for cartilage regeneration.

## Figures and Tables

**Table 1:** The different types of signal plus their functionalities and crucial roles in the maintenance of cartilage homeostasis [43].

Type of Signal	Role (Duty)
TGF-β	Regulates the proliferation/differentiation of chondrocytes, and stimulates the RY-box expression
BMP	Plays an important role in various stages of skeletal growth, and the commitment of mesenchymal cells to the lines of chondrocytes in the induction of proliferation and cell maturation in the growth and formation of the joints and bones
IGF	Develops cartilage and reproduces chondrocytes on the growth plate
FGF	Develops vital organs

**Table 2:** Various cellular signaling pathways and their significant role in cartilage tissue engineering.

Signaling Pathway	Role (Duty)
SMAD	Expresses pre-hypertrophic and proliferative and hypertrophic chondrocytes in all regions of the cartilage
b-catenin-dependent	Stimulates bone growth in axial growth, and induces endochondral ossification and axial growth
Non-canonical WNT	Creates growth pillars by chondrocytes

**Table 3:** A brief view at the mechanical properties, concentrations, and the methods of applications of agarose-based biomaterials in biomedical applications.

Material	Concentration %	Mechanical Properties (KPa)	Methods	Action	Ref.
Agarose	1	10	The effect of TGF-b3 was compared to the fatal bovine serum. Mechanical properties were assessed at day 42.	Low-concentration agarose stimulates the formation of more Homogeneous ECM distribution. The presence of TGF-b3 is also beneficial because it stimulates the distribution of matrix components. Both stimuli result in constructs with improved mechanical properties	[60]
Agarose	2	16			
Agarose	3	35			
Agarose-FBS	1	15			
Agarose-FBS	2	25			
Agarose-FBS	3	40			
Agarose-TGF	1	40			
Agarose-TGF	2	45			
Agarose-TGF	3	50			
Agarose-TGF	0.25	40			
Agarose-TGF	0.5	27			
Agarose-TGF	1	34			
Agarose-TGF	1 disc	38			
Agarose	2	1.547	Gel	Both the volume fraction of water and hydraulic permeability decreased with increasing agarose gel concentration. Permeability was dependent on hydrogel and cartilage deformation	[78]
	2.5	4.237			
	3	10.03			
	4	10.55			
	6	33.16			
	10	82.34			
	14.8	333.4			
PRP-agarose	7 days 14 days	32 50	Porcine chondrocytes were seeded in agarose gel and platelet-rich plasma–agarose gel.	The hydrogel possesses a proper microenvironment for chondrocyte growth, proliferation and matrix formation	[79]
	28 days	75			
AG-BM Strong modulus		13.20	Blended hydrogels	The degradation capability of blended hydrogel circumvents the drawbacks of the otherwise non-degradable pure agarose The hydrogels with non-mulberry SF blends showed larger pore size as compared to the mulberry blend hydrogels. The rheological studies revealed elasticity of the blended hydrogels had a yield point at a higher amplitude strain as compared to pure agarose	[53]
AG-BM Loss modulus		4.96			
		
		
			Controlled emulsion technique		[80]
